# Luminescence Properties of Hoechst 33258 in Polyvinyl Alcohol Films

**DOI:** 10.3390/ijms26020514

**Published:** 2025-01-09

**Authors:** Bong Lee, Agnieszka Jablonska, Danh Pham, Rajveer Sagoo, Zygmunt Gryczynski, Trang Thien Pham, Ignacy Gryczynski

**Affiliations:** Department of Physics and Astronomy, Texas Christian University, Fort Worth, TX 76129, USA; a.m.jablonska@tcu.edu (A.J.); danh.pham@tcu.edu (D.P.); rajveer.sagoo@tcu.edu (R.S.); z.gryczynski@tcu.edu (Z.G.);

**Keywords:** Hoechst, room temperature phosphorescence, direct triplet excitation, long-lived phosphorescence

## Abstract

We report a comprehensive investigation of the photophysical properties of Hoechst 33258 (HOE) embedded in polyvinyl alcohol (PVA) films. HOE displays a bright, highly polarized, blue fluorescence emission centered at 430 nm, indicating effective immobilization within the polymer matrix of PVA. Its fluorescence quantum yield is notably high (~0.74), as determined relative to a quinine sulfate standard. In addition, we observed that HOE-doped PVA films exhibit room temperature phosphorescence (RTP) that remains visible for several seconds after UV excitation ceases. The slightly negative phosphorescence anisotropy implies that the triplet–singlet radiative transition is orthogonal to the singlet–singlet transition governing fluorescence. Notably, we observed that direct triplet-state excitation at longer wavelengths (beyond the primary absorption band) produces highly polarized RTP. We believe this possibility of direct triplet-state excitation opens new avenues for studying RTP in polymer-immobilized molecules.

## 1. Introduction

Hoechst 33258 (HOE), a bisbenzimide derivative, was first synthesized by Hoechst AG in the early 1970s [1,2]. This fluorescent dye has since become a widely used tool for deoxyribonucleic acid (DNA) staining in various scientific applications [3]. It selectively binds to the minor groove of AT-rich double-stranded DNA, exhibiting a blue fluorescence emission upon ultraviolet (UV) excitation [4,5,6]. Notably, Hoechst dyes are commonly used as substitutes for DAPI [7]. While they share DAPI’s high specificity for DNA and preference for A-T base pairs, Hoechst dyes offer advantages such as lower cytotoxicity and greater binding flexibility [8]. Consequently, Hoechst dyes are often preferred for staining DNA in both living and fixed cells. The fluorescent properties of Hoechst dyes allow for their use in fluorescence microscopy, flow cytometry, and immunochemistry [9,10]. Recent studies have expanded their application to developing composite probes, intracellular targeting, and creating new molecular sensors [11,12,13,14,15]. Moreover, Hoechst dyes’ ability to visualize and quantify cellular DNA makes them useful in advanced imaging techniques, drug delivery, and live-cell imaging studies [16,17,18,19]. These attributes make Hoechst dyes a valuable tool in contemporary biomedical research.

Despite its wide range of applications, the room temperature phosphorescence (RTP) of Hoechst 33258 has not been explored. RTP offers distinct advantages compared to fluorescence, including significantly larger Stokes shifts, which allow for better spectral separation of excitation and emission wavelengths. Additionally, RTP features longer emission lifetimes, which facilitate time-resolved measurements and reduce background interference, ultimately leading to enhanced signal-to-noise ratios and improved detection sensitivity [20,21,22,23]. These properties have made RTP increasingly attractive for various applications such as encryption [24,25], decryption [26], anticounterfeiting [27], and bioimaging [28]. Most frequently, RTP materials are inorganic or organometallic [29]. However, these materials often face limitations due to their scarcity, high cost, and cytotoxicity [30,31]. Consequently, researchers have increasingly turned their attention to pure organic RTP materials, which offer flexible molecular designs and tunable properties [32].

Phosphorescence involves the transition from the lowest singlet state (S_1_) to the excited triplet state (T_n_) through intersystem crossing (ISC), and then returning to the ground state (S_0_) [33]. For RTP to be effective, two conditions must be met: first, the triplet excited state must be efficiently populated from S_1_ to T_n_ through ISC. Strong spin–orbit coupling (SOC) is essential for facilitating this process. Second, it is crucial to suppress non-radiative deactivation pathways in the triplet excited state to prevent quenching by external factors such as oxygen and humidity. This can be achieved by reducing the thermal motion of molecules through methods such as crystallization, polymerization, and matrix rigidification [33,34,35,36].

Poly vinyl alcohol (PVA) films meet such criteria and have emerged as a prominent polymer matrix for RTP studies due to their excellent film-forming properties, biocompatibility, and ease of processing [37,38,39,40]. Our laboratory, along with the broader scientific literature, has reported RTP of small organic molecules embedded in PVA films [41,42,43,44,45,46]. Additionally, PVA’s compatibility with a wide range of phosphorescent materials underscores its versatility and effectiveness in RTP applications [47,48,49,50,51,52,53,54,55]. For instance, Chen et al. reported on aggregation-induced RTP from self-quenching-resistant nitrogen-doped carbon dot powder within PVA, taking advantage of its moisture-resistant properties [56]. Moreover, the RTP of metal–organic hybrids has been documented, with Yang et al. reporting RTP from the embedding of a Zn-IPA Metal–Organic Framework (MOF) into a PVA matrix [48]. Recently, Zeonex, an excellent polymer matrix and superior optical material, has been used in RTP [57,58,59,60].

Hence, in this study, we investigate the photophysical properties of Hoechst 33258 in PVA films. Building on our previous work with DAPI-doped PVA, which demonstrated the potential for RTP [61], we posit that the matrix rigidification afforded by embedding Hoechst or DAPI in polymeric films may extend to biological matrices [4,5,6]. Although RTP has been achieved in various aqueous solutions, the use of naturally occurring biological structures, such as DNA, as a matrix remains largely unexplored. Past efforts have instead relied on inorganic substances (e.g., quaternary ammonium) or techniques (e.g., silica encapsulation) to ensure robust matrix formation [62,63,64]. Notably, leveraging DNA as a matrix for RTP could open new avenues for applications beyond simple DNA detection—particularly in advanced biomedical imaging techniques—by offering highly stable, low-toxicity systems with potentially longer-lived phosphorescent signals.

## 2. Results

### 2.1. Absorption and Fluorescence Spectra

**Figure 1:**
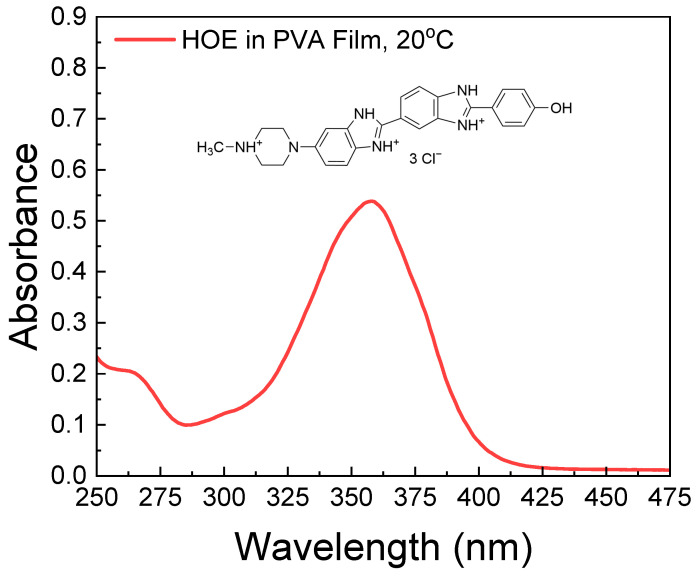
Absorption spectrum of HOE-doped PVA film at 20 °C. The chemical structure of HOE is shown above the spectrum.

The absorption spectrum of HOE-doped PVA film is shown in Figure 1. It consists of a long-wavelength band with a peak absorption corresponding to the S_0_–S_1_ transition at ~350 nm and weaker peaks below 310 nm corresponding to higher excited states.

By taking into account the extinction coefficient of HOE at its maximum peak (47,000 M^−1^ cm^−1^) [65], and the thickness of the film (200 μm), the concentration of HOE used in the film is approximately 0.5 mM. This concentration is optimal for absorption measurements and provides an excellent signal-to-noise ratio for emission measurements. We prepared a series of films with absorbances from 0.1 to 1.0 (corresponding to concentrations from 0.1 mM to 1 mM) and observed no differences in the shapes of the spectra. This indicated the absence of HOE aggregates in the films.

**Figure 2:**
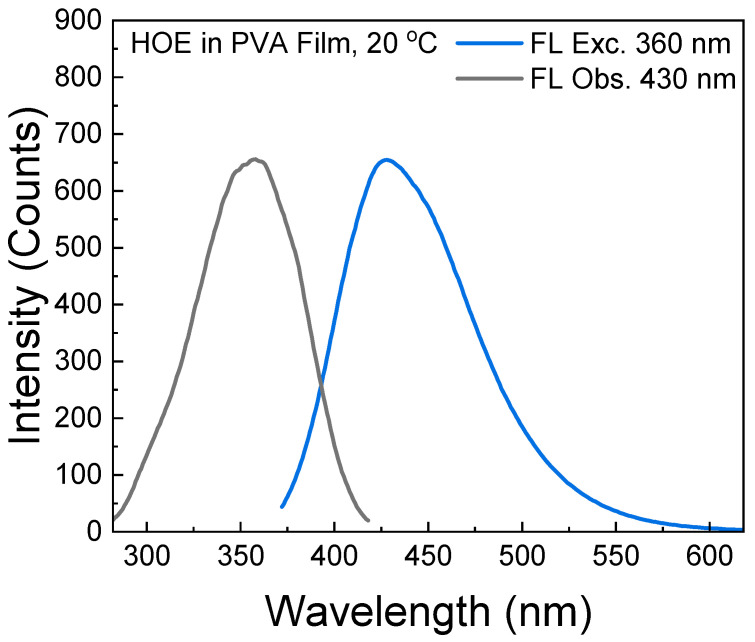
Excitation and emission fluorescence spectra of HOE-doped PVA film.

The fluorescence excitation and emission spectra of HOE-doped PVA film are shown in Figure 2, where the excitation spectrum matches the absorption spectrum. The bright blue fluorescence emission spans from 375 nm to 575 nm with a peak at 430 nm. The small spectral overlap (Stoke’s shift) of HOE-doped PVA prevents the excitation energy migration process which may lower fluorescence anisotropy.

### 2.2. Fluorescence Anisotropy

**Figure 3:**
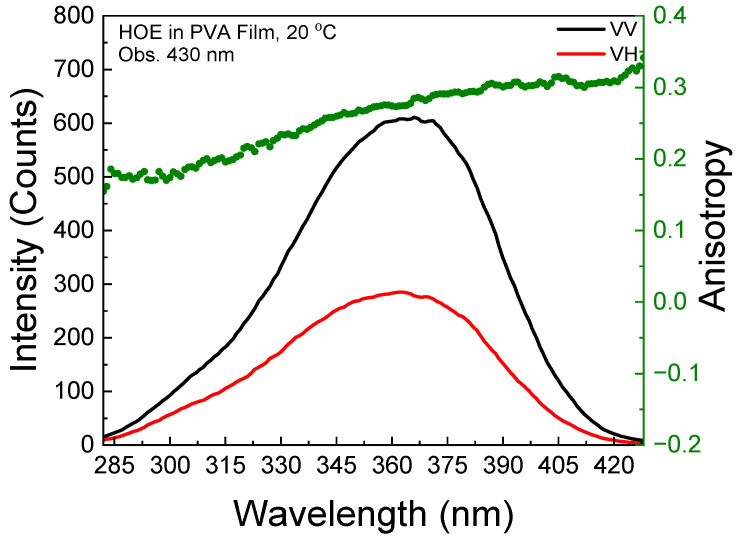
Fluorescence excitation anisotropy spectrum of HOE-doped PVA film (green dots correspond to anisotropy values).

**Figure 4:**
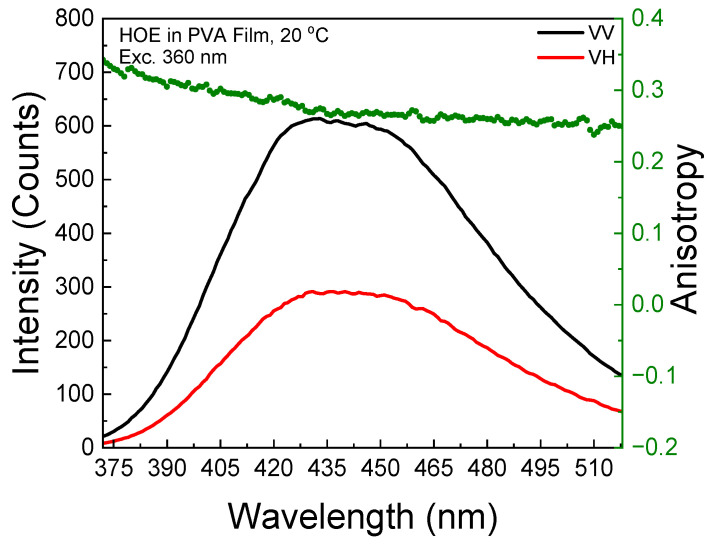
Fluorescence emission anisotropy spectrum of HOE-doped PVA film (green dots correspond to anisotropy values).

The polarized components from the fluorescence excitation spectra of HOE-doped PVA film, along with the anisotropy values calculated using Equation (1), are shown in Figure 3. At longer excitation wavelengths, the anisotropy reaches 0.3, indicating that the HOE molecules are immobilized. The high value of the fluorescence anisotropy spectrum is also shown in the fluorescence emission; see Figure 4.

### 2.3. Fluorescence Quantum Yield

**Figure 5:**
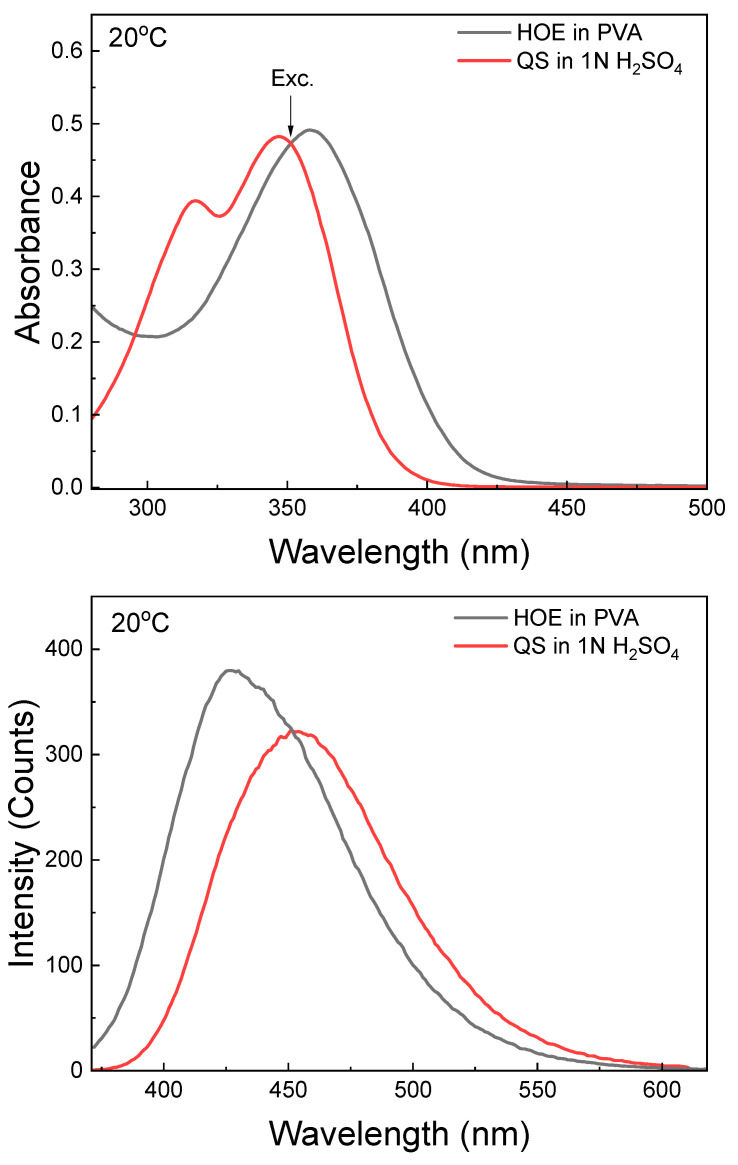
Comparison of fluorescence emissions of HOE in PVA and quinine sulfate in 1N H_2_SO_4_. Top: absorbances; bottom: fluorescence intensities. The excitation was at equal absorbances at 351 nm.

The fluorescence efficiency of HOE-doped PVA film was estimated using quinine sulfate (QS), a commonly used quantum yield (QY) standard. QS was dissolved in 1N H_2_SO_4_ and the solution was placed in a 1 mm thick microcuvette. A strip of HOE-doped PVA film was placed into the microcuvette and filled with benzene to match the refractive index of PVA. Both absorption and fluorescence spectra of QS matched those of HOE. With the excitation set at 351 nm, where absorbances of QS and HOE are identical (see Figure 5, top), the QY of HOE-doped PVA film was calculated as a product of integrated intensities ratio and squares of the ratio of refractive indexes (HOE/QS); see details in the Section 3. The QY of HOE-doped PVA was estimated to be 0.74.

### 2.4. Fluorescence Lifetime

**Figure 6:**
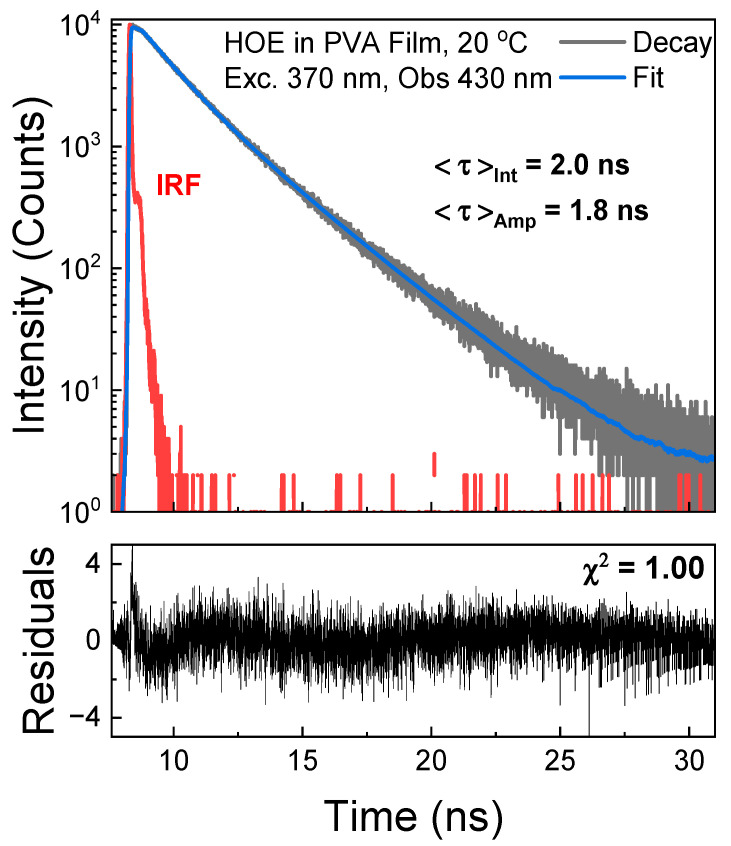
Fluorescence intensity decay of HOE-doped PVA film. The decay was fitted to a double exponential model with recovered parameters $\tau_{1}=1.27\;ns$ and $\tau_{2}=2.58\;ns$, and amplitudes $\alpha_{1}=0.58$ and $\alpha_{2}=0.42$. Instrument response function (IRF) is in red.

The fluorescence intensity decay of HOE-doped PVA was measured in time-domain mode with an excitation from a pulsed 370 nm laser diode and observation set at 430 nm. The decay was approximated with two exponents yielding an average lifetime of 2 ns; see Figure 6.

### 2.5. Phosphorescence Spectra

**Figure 7:**
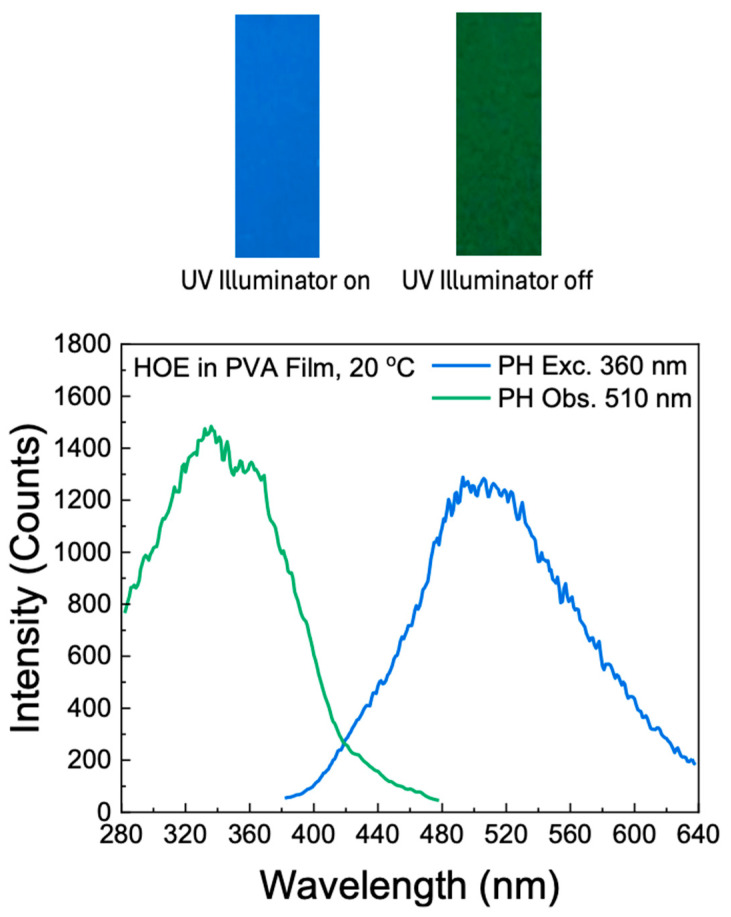
Top: a strip of HOE-doped PVA film on the UV illuminator; bottom: phosphorescence excitation and emission spectra of HOE-doped PVA film.

The RTP of HOE-doped PVA was measured with the gating parameters described in the Section 3. The RTP of the HOE-doped PVA film was green and persisted for a couple of seconds after being exposed on the UV illuminator; see Figure 7, top. The RTP spectrum spans from 400 nm to about 650 nm; see Figure 7. The RTP excitation spectrum differs from the fluorescence excitation spectrum; compare Figure 7 and Figure 2. First, its maximum is shifted towards shorter wavelengths. Second, unlike the fluorescence, the phosphorescence can be excited at longer wavelengths up to 480 nm, suggesting the possibility of direct triplet-state excitation. The illumination at wavelengths longer than 420 nm primarily excites the triplet state.

### 2.6. Phosphorescence Lifetime

**Figure 8:**
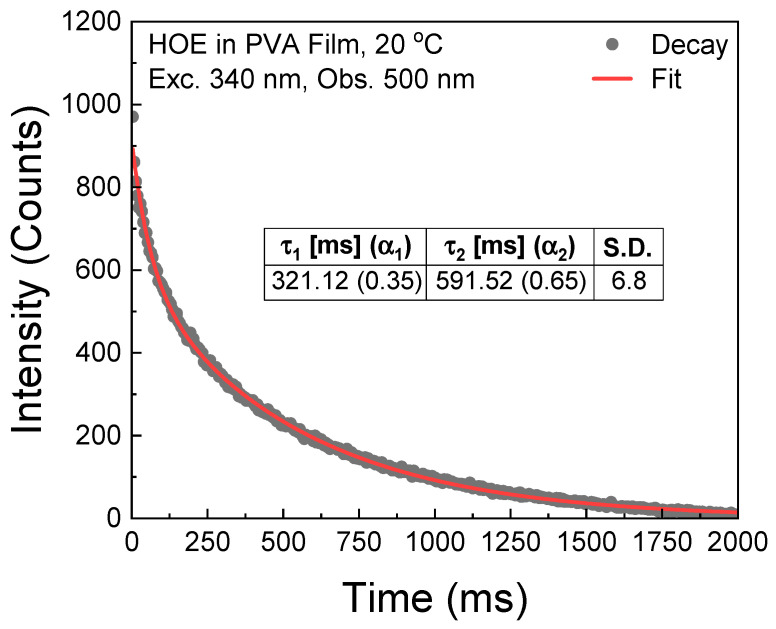
Phosphorescence intensity decay of HOE-doped PVA film.

The phosphorescence lifetime of the HOE-doped PVA film was measured with the Varian Eclipse spectrofluorometer in phosphorescence lifetime mode with gating parameters detailed in the Section 3. The phosphorescence intensity decay was fitted with two exponents; see Figure 8.

### 2.7. RTP Anisotropy

**Figure 9:**
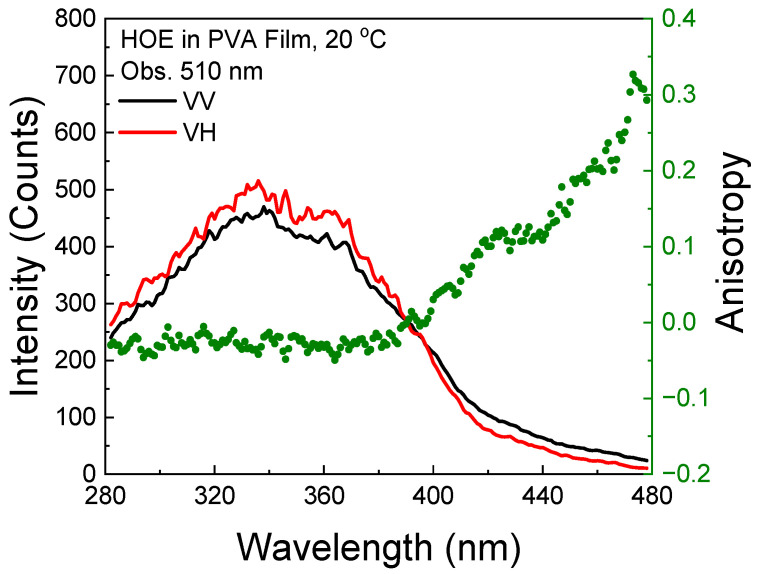
Phosphorescence excitation anisotropy spectrum of HOE-doped PVA film (green dots correspond to anisotropy values).

The phosphorescence excitation anisotropy is slightly negative within the S_0_–S_1_ absorption range as expected since the phosphorescence transition is orthogonal to the absorption/fluorescence transition. However, above 400 nm, there is a sharp increase, indicating the direct excitation to the triplet state; see Figure 9.

**Figure 10:**
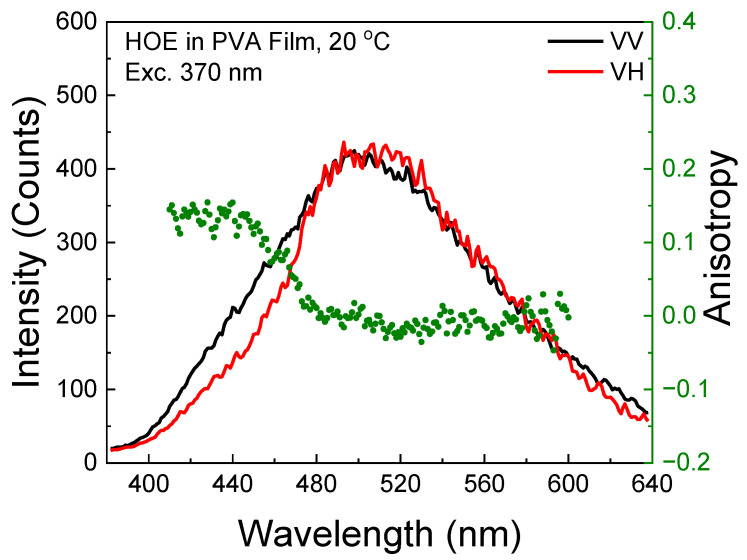
Phosphorescence emission spectrum of HOE-doped PVA film (green dots correspond to anisotropy values).

The phosphorescence emission anisotropy exhibited slightly higher values at shorter observation wavelengths, indicating the presence of residual delayed fluorescence; see Figure 10. In the phosphorescence wavelength region, the anisotropy is slightly negative.

### 2.8. RTP with Direct Triplet-State Excitation

**Figure 11:**
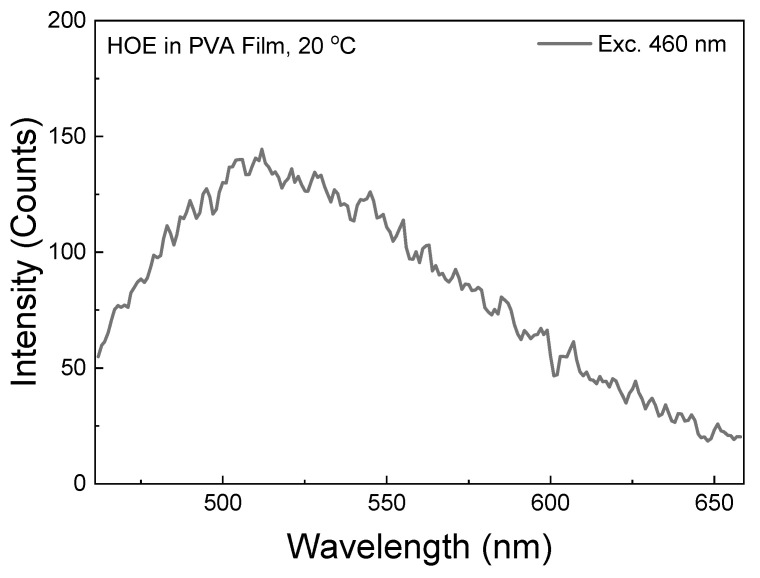
RTP emission spectrum of HOE-doped PVA measured with 460 nm excitation. The gating parameters were the same as for other RTP measurements.

**Figure 12:**
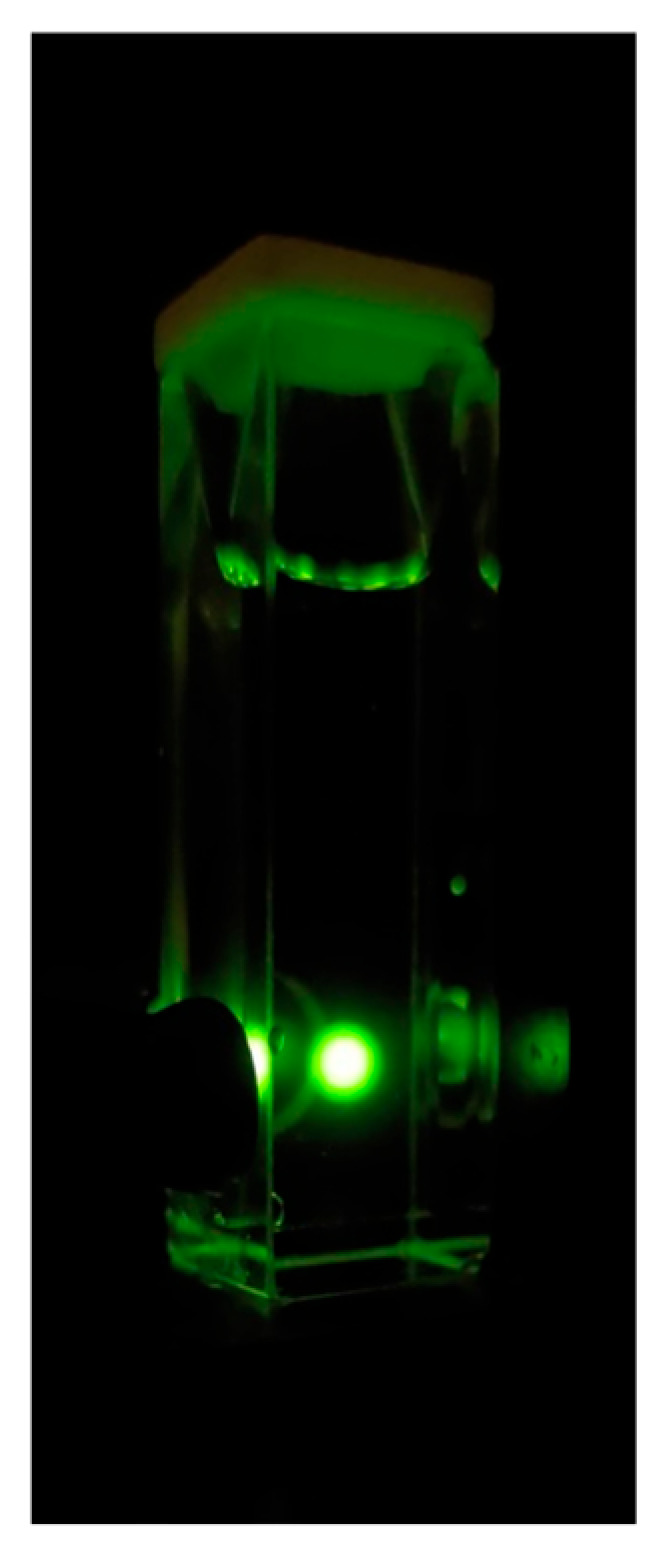
HOE-doped PVA film phosphorescence upon 480 nm excitation from a laser diode.

The RTP spectrum with an excitation of 460 nm (well outside of HOE-doped PVA film absorption band; see Figure 1) is shown in Figure 11. Although the spectrum is noisy as it is measured at the limit of the instrument sensitivity, it does not involve the intersystem crossing process. With a stronger excitation from a 480 nm laser diode, the RTP becomes bright and easily visible to the naked eye; see Figure 12.

## 3. Materials and Methods

The bisbenzimide (Hoechst 33258) was from Molecular Probes (Eugene, OR, USA). Polyvinyl alcohol (PVA) [MW 130,000, 98% hydrolyzed] was obtained from Sigma-Aldrich (St. Louis, MO, USA).

### 3.1. Film Preparation

A bulk solution was prepared in a 1 L Erlenmeyer flask using a 10% (*w*/*w*) PVA solution in deionized water. The solution was heated and stirred at 95 °C until the PVA completely dissolved and reached a honey-like consistency. Then, it was allowed to cool. A small volume of Hoechst 33258 stock solution was added to 20 mL of PVA solution, throroughly mixed, and then poured into an 85 mm Petri dish. Blank PVA films were made using a similar method to serve as control for background signal correction. The PVA films were set aside to dry for approximately one week. Once the films were dried, a razor blade was used to peel the PVA films from the Petri dishes. Using calipers, the thicknesses of the PVA films were measured to be ~200 microns.

### 3.2. Absorption Spectra

The Varian Cary 60 UV-Vis Spectrophotometer (Agilent Technologies, Inc., Santa Clara, CA, USA) was used for the room temperature absorption measurements. Unless otherwise specified, the absorbance spectra were corrected using the blank PVA film as a baseline.

### 3.3. Fluorescence Spectra

The Varian Cary Eclipse Spectrofluorometer (Agilent Technologies, Inc., Santa Clara, CA, USA) was used to conduct steady-state fluorescence measurements. A front-face configuration was achieved using a custom attachment on the Varian Cary Eclipse spectrofluorometer [66]. Anisotropy measurements were performed using a UV grid polarizer and a plastic sheet polarizer added to the excitation and emission sides, respectively.

### 3.4. Fluorescence Anisotropy

The fluorescence excitation and emission anisotropies were calculated from the measured polarized intensity components I_VH_ and I_VV_ as

(1) $$r=\frac{I_{VV}-I_{VH}*G}{I_{VV}+2I_{VH}*G}$$

where the I_VV_ and I_VH_ components are the fluorescence intensities when excited with vertical polarization (V) and observed with horizontal (H) or vertical polarization, respectively. G (G-Factor) was used to account for the uneven transmissions of I_VH_ and I_VV_ components through the detection path. Equation (1) was utilized for both phosphorescent and fluorescent emission measurements.

### 3.5. Fluorescence Quantum Yield

To estimate the fluorescence quantum yield (QY), the HOE sample was compared to a solution of quinine sulfate (QS) in 1N H_2_SO_4_—a well-established QY standard (QY = 0.54 [67]). The measurements were conducted using a front-face configuration with 1 mm path-length microcuvettes. Benzene was added to the cuvette containing the HOE-doped PVA film as it has the same refractive index as the PVA film. The different refractive indices for QS in 1N H_2_SO_4_ (n = 1.35) and for HOE-doped PVA film (n = 1.48) were accounted for in the QY calculation.

### 3.6. Fluorescence Lifetime

Fluorescence lifetime measurements were conducted using an FT200 fluorometer (PicoQuant GmBH, Berlin, Germany), which is equipped with a time-correlated single photon counting module (PicoHarp 300, PicoQuant GmBH, Berlin, Germany) and with an ability to resolve 4 ps. A 370 nm pulsed laser diode (LDH-P-C-375B, PicoQuant GmBH, Berlin, Germany) was used with a repetition rate of 4 MHz (PDL 800-B, PicoQuant GmBH, Berlin, Germany). The time-dependent data were analyzed using FluoFit4 (PicoQuant GmBH, Berlin, Germany).

### 3.7. Phosphorescence Spectra Measurements

Phosphorescence emission and excitation spectra were measured with the Varian Cary Eclipse using its time-gated phosphorescence detection mode. This setting eliminates short-lived emission components, including but not limited to Raman scattering, Rayleigh scattering, and fluorescence background signals. Unless otherwise specified, the parameters used in this setting were as follows: Total Decay Time—1.0 s, Number of flashes—10, Delay Time—0.5 ms, and Gate Time—5 ms. The phosphorescence intensity decay was measured up to 2 s. The total phosphorescence excitation and emission anisotropies were calculated from the measured polarized phosphorescence intensity components I_VH_ and I_VV_, as described by Equation (1).

## 4. Conclusions

In this study, we comprehensively characterized the fluorescence and phosphorescence properties of HOE-doped PVA films. The bright, highly polarized, blue fluorescence (peaking at 430 nm) is associated with a notably high quantum yield (~0.74), indicating the effective immobilization of HOE within the polymer matrix. Time-resolved measurements revealed a relatively short fluorescence lifetime of ~2 ns, whereas RTP displayed a longer lifetime of ~0.5 s. When excited within HOE’s primary absorption band, the phosphorescence showed slightly negative anisotropy, suggesting that the triplet–singlet radiative transition is orthogonal to the singlet–singlet transition. Notably, we observed that long-wavelength excitation beyond the main absorption band can directly populate the triplet state, producing a highly polarized phosphorescence similar to that of fluorescence. The RTP brightness of HOE-doped PVA film samples is relatively high, comparable to DAPI and other reported organic compounds. With a long-wavelength excitation outside the main absorption band, stronger excitations from lasers can be used to obtain a very high RTP emission, as shown in Figure 12. We believe that this possibility of a direct triplet-state excitation route broadens the potential for novel spectroscopic and imaging applications of HOE.

## Data Availability

The data presented in this study are available on request from the corresponding authors. The data are not publicly available due to privacy.
